# Myeloid-associated B-cell identity remodeling in CAR T-cell resistance: a hypothesis-generating framework for diffuse large B-cell lymphoma

**DOI:** 10.3389/fimmu.2026.1895943

**Published:** 2026-08-21

**Authors:** Gaojie Liu, Junwei Yang, Han Li, Jingyi Bao, Liuxin Jiang, Lishuo Deng, Yuetong Tan, Yuxiang Song, Yao Wang

**Affiliations:** 1 Department of Gastrointestinal Surgery, The Affiliated Cancer Hospital, Guangzhou Medical University, Guangzhou, Guangdong, China; 2 Guangzhou Institute of Cancer Research, The Affiliated Cancer Hospital, Guangzhou Medical University, Guangzhou, Guangdong, China; 3 College of Mathematics and Statistics, Hubei Normal University, Huangshi, Hubei, China; 4 Department of Medical Oncology, The Affiliated Cancer Hospital, Guangzhou Medical University, Guangzhou, Guangdong, China

**Keywords:** diffuse large B-cell lymphoma, large B-cell lymphoma, CAR T-cell resistance, CD19, B-cell identity, tumor microenvironment, myeloid cells, antigen escape

## Abstract

CD19-directed chimeric antigen receptor (CAR) T-cell therapy has transformed the treatment of relapsed or refractory large B-cell lymphoma (LBCL), including diffuse large B-cell lymphoma (DLBCL), yet primary resistance and relapse remain frequent. Clinical studies link pretreatment B-cell gene expression signatures, *CD19* gene expression, malignant-cell CD19 protein expression, immune contexture and myeloid/inflammatory features with CAR T-cell outcomes, but they do not establish that myeloid cells directly induce loss of B-cell identity. In this Mini Review, we use B-cell identity remodeling as a DLBCL-focused, hypothesis-generating research construct describing concordant quantitative or spatially heterogeneous attenuation across B-lineage antigens and transcriptional programs without requiring frank lineage conversion; isolated CD19 attenuation alone is considered antigen attenuation rather than sufficient evidence of broader identity remodeling. We explicitly distinguish direct clinical associations derived largely from broader LBCL cohorts, indirect biological support from DLBCL single-cell and spatial studies, and untested mechanisms through which myeloid or stromal signals might select, stabilize or protect B-cell-identity-low states. Proposed biomarkers are framed as candidate research variables rather than validated clinical thresholds, with PAX5 distinguished from the more context-dependent role of IRF8. Finally, therapeutic hypotheses are organized by antigen dependence and biological compartment, and separate workflows are outlined for mechanistic discovery, prospective translational testing and future clinical implementation. This framework is intended to sharpen testable questions rather than to claim an established causal myeloid-B-cell identity axis.

## Highlights

- The framework links malignant-cell targetability with myeloid/stromal context while explicitly separating clinical association from mechanistic hypothesis.
- In LBCL CAR T-cell cohorts, B-cell gene expression signatures, *CD19* transcript abundance and malignant-cell CD19 protein expression are associated with outcome; lower malignant-cell CD19 protein expression also associates with immunosuppressive stromal/myeloid features, without establishing causality.
- DLBCL single-cell studies define heterogeneous tumor and immune states, whereas spatial studies demonstrate organized tumor-immune niches; together, they provide indirect biological support for testing local ecological interactions in CAR T-cell resistance.
- B-cell identity remodeling is treated as a multivariable research construct: isolated CD19 attenuation alone is not sufficient, and PAX5 is distinguished from the more context-dependent role of IRF8.
- Therapeutic hypotheses are organized by antigen dependence and biological compartment, separating alternative-antigen targeting, tumor-cell antigen modulation, microenvironmental remodeling and CAR T-cell fitness.

## 1 Introduction

Diffuse large B-cell lymphoma (DLBCL) is the most common aggressive B-cell non-Hodgkin lymphoma and is heterogeneous at genetic, transcriptional and microenvironmental levels (1–6). CD19-directed CAR T-cell therapy has transformed the treatment of relapsed or refractory large B-cell lymphoma (LBCL), with durable remissions in subsets of patients treated with axicabtagene ciloleucel, tisagenlecleucel or lisocabtagene maraleucel (7–9). In the second-line setting, axi-cel improved outcomes compared with standard salvage therapy in ZUMA-7 (10, 11). However, primary resistance and relapse remain frequent.

CAR T-cell resistance is commonly considered in three compartments: CAR T-cell-intrinsic factors, tumor-intrinsic factors and the tumor microenvironment (TME). These include inadequate expansion or persistence, antigen loss or low antigen density, apoptosis resistance, suppressive myeloid populations, stromal barriers, inflammatory signaling and impaired immune trafficking (12–18). These compartments are analytically useful, but clinical data suggest that malignant-cell targetability and immune contexture may coexist rather than operate independently.

This Mini Review focuses biologically on DLBCL and uses B-cell identity remodeling as a research construct describing quantitative or spatially heterogeneous attenuation of B-lineage antigens and transcriptional programs without requiring frank lineage conversion. The construct is intended to organize candidate measurements and testable hypotheses, not to define a validated diagnostic entity.

Several pivotal CAR T-cell studies discussed here enrolled broader LBCL populations that included histologies beyond DLBCL. We therefore identify findings from mixed LBCL cohorts as LBCL-derived and do not treat them as DLBCL-specific unless subgroup evidence is available. We also distinguish three evidence levels. Direct clinical evidence from CAR T-cell-treated LBCL cohorts links B-cell gene expression signatures (B-cell GES), *CD19* gene expression, malignant-cell CD19 protein expression and myeloid/stromal or inflammatory features with outcome. Single-cell and spatial studies in DLBCL provide indirect biological support for organized immune niches but generally do not test CAR T-cell resistance in paired longitudinal samples. Finally, the proposition that myeloid-derived signals induce, stabilize or protect B-cell-identity-low malignant states remains a mechanistic hypothesis requiring longitudinal sampling and functional perturbation. The framework is motivated particularly by clinical observations from ZUMA-7 linking B-cell GES, *CD19* expression, malignant-cell CD19 protein and stromal/myeloid features with axi-cel outcomes (12); these associations are discussed in detail below.

## 2 CAR T-cell resistance across LBCL: relevance to DLBCL

CD19 is expressed across most B-cell malignancies, including DLBCL, but CD19-directed therapy imposes strong selection pressure. Relapse may occur with absent or reduced CD19, whereas other tumors remain CD19 positive but resist therapy through impaired CAR T-cell persistence, poor infiltration, inhibitory signaling or tumor-cell survival programs (16–18). Binary CD19 positivity therefore does not fully capture antigen density, spatial heterogeneity or the stability of broader B-lineage programs.

Lineage switching after CD19-directed therapy is well described in B-cell acute lymphoblastic leukemia (16, 18), but this phenomenon should not be assumed to occur similarly in DLBCL. For DLBCL, the framework considered here is more limited: malignant cells may retain lymphoma morphology and substantial B-cell features while showing quantitative or regional attenuation of B-lineage antigens or transcriptional programs. This proposed state should be distinguished from plasmacytic differentiation and from convincing myeloid or other cross-lineage conversion.

Accordingly, targetability can be considered as a continuum for research purposes. Tumors with preserved CD19 and B-lineage features may fail predominantly because of CAR T-cell or TME constraints, whereas tumors with low or heterogeneous antigen density may be less efficiently recognized. Multi-antigen approaches can reduce escape caused by isolated loss of one target (19, 20), but may be insufficient if several B-lineage antigens are attenuated together. This possibility motivates direct measurement of antigen density and lineage-associated programs rather than inference from routine CD19 positivity alone.

## 3 Candidate research variables for B-cell identity remodeling

We use B-cell identity remodeling as a research construct rather than a validated diagnostic category. Candidate variables include continuous measures of a prespecified B-cell GES, malignant-cell CD19 protein density, *CD19* transcript abundance, additional B-lineage antigens and lineage-associated regulators. Importantly, isolated reduction of CD19 alone should be described as antigen attenuation rather than B-cell identity remodeling; the latter construct is intended for concordant attenuation across broader B-lineage transcriptional or phenotypic features. B-cell GESs should not be assumed to be interchangeable across platforms: future studies should prespecify gene composition, assay platform, normalization and scoring method. No cross-platform or clinically validated cutoff currently exists.

PAX5 provides a well-established lineage-maintenance anchor for this construct. Experimental studies show that PAX5 maintains B-cell identity by activating B-cell-specific genes and repressing lineage-inappropriate programs (21–23). Reduced PAX5 activity may therefore be mechanistically informative when interpreted with antigen and transcriptional readouts. IRF8 should be treated differently. Although IRF8 participates in B-cell biology and lymphoma immune regulation, including MHC class II antigen processing, current evidence does not establish reduced IRF8 as a surrogate for coordinated attenuation of CD19, CD20 and CD22 (24, 25). Resistance-associated alterations involving *PAX5* and *IRF8* have also been reported in engineered T-cell therapy datasets, but these observations do not establish a myeloid upstream driver (15). IRF8 is therefore better considered an exploratory contextual marker than an equivalent core identity marker.

Thresholds based on quartiles, immunohistochemical H-scores or flow-cytometric mean fluorescence intensity are assay-, tissue- and cohort-dependent. Future studies may use such thresholds for within-cohort hypothesis testing, but they should not be interpreted as diagnostic criteria or routine treatment-selection rules. Sampling time should also be specified because archival diagnostic tissue, post-bridging or pre-lymphodepletion tissue, early on-treatment specimens and relapse biopsies represent biologically non-equivalent time points and should not be pooled without explicit justification.

The proposed construct also requires exclusion of alternative biological explanations. Plasmacytic differentiation may reduce PAX5 and CD20 protein while increasing plasma-cell markers such as BLIMP1, CD38 and CD138, together with activation of *PRDM1* and *XBP1* transcriptional programs. True myeloid transdifferentiation requires convincing morphologic and lineage evidence. The state considered here therefore refers only to DLBCL cells that retain lymphoma identity while showing broader reduction or heterogeneity of B-lineage antigens and transcriptional features. Candidate research variables, their evidence status, and key interpretive caveats are summarized in **Table 1**.

**Table 1:** Candidate research variables for evaluating B-cell identity remodeling.

Domain	Candidate research readouts	Evidence status	Key caveat
Lineage program	Prespecified B-cell GES; PAX5 protein or *PAX5* transcript; BCR-related transcripts	Candidate research dimension; continuous measures preferred	Specify signature genes, platform, normalization and scoring; no validated cross-platform cutoff
CD19 targetability	CD19 protein density; IHC H-score or flow-cytometric MFI; *CD19* transcript abundance	Measures target density for CD19-directed recognition	Thresholds are assay-, tissue- and cohort-dependent; assign signal to malignant cells
Additional B-lineage antigens	CD20, CD22 and CD79A/CD79B protein; *MS4A1, CD22, CD79A* and *CD79B* transcript abundance	Tests whether attenuation extends beyond CD19	Isolated CD19 loss is antigen attenuation; coordinated reduction does not establish lineage switch
Contextual regulator	IRF8 protein or *IRF8* transcript	Exploratory contextual marker	Not established as an equivalent surrogate for coordinated CD19/CD20/CD22 attenuation
Spatial context and exclusions	Myeloid/stromal colocalization; plasma-cell markers and morphology	Tests niche association and alternative differentiation states	Spatial association is not causation; exclude plasmacytic or cross-lineage conversion

BCR, B-cell receptor; GES, gene expression signature; IHC, immunohistochemistry; MFI, mean fluorescence intensity; MDSC, myeloid-derived suppressor cell; TAM, tumor-associated macrophage.

## 4 Clinical evidence from LBCL cohorts and biological support from DLBCL studies

Clinical evidence directly linking myeloid features to CAR T-cell outcome is strongest at the level of association. Jain and colleagues reported that pretreatment tumor interferon signaling, high blood levels of monocytic myeloid-derived suppressor cells (M-MDSCs), and elevated blood IL-6 and ferritin were each associated with failure to achieve a durable response to CD19-directed CAR T-cell therapy in LBCL (14). In ZUMA-1 samples, pretreatment immune contexture measured by Immunoscore and Immunosign 21 was associated with clinical response and overall survival after axi-cel (13). These studies support the relevance of the pretreatment immune environment but do not demonstrate coordinated B-lineage remodeling.

The ZUMA-7 exploratory analysis provides an important clinical bridge between tumor-cell targetability and stromal/myeloid contexture. The prespecified NanoString B-cell GES and bulk-tumor *CD19* gene expression were associated with axi-cel outcome; malignant-cell CD19 protein H-score correlated with *CD19* gene expression and was also associated with outcome. Lower malignant-cell CD19 protein expression was associated with a tumor GES enriched for immunosuppressive stromal and myeloid genes (12). Importantly, axi-cel remained superior to standard of care across high and low biomarker subgroups, underscoring that neither binary CD19 status nor any proposed identity threshold is currently suitable as a treatment-selection rule. These observations establish association and co-occurrence, not direct myeloid suppression of CD19 or causation of B-cell identity loss.

DLBCL-specific single-cell and spatial studies provide a different level of evidence. They demonstrate heterogeneous tumor-cell states, structured immune ecosystems and spatially defined inflammatory or macrophage-rich niches (26–30). Broader B-cell lymphoma studies likewise support clinically meaningful microenvironmental heterogeneity (31). These findings make local ecological interactions biologically plausible, but most were not designed around CAR T-cell resistance and did not include paired pretreatment and post-CAR T-cell specimens. They therefore provide indirect biological support rather than direct evidence for the proposed axis.

Taken together, available data justify testing whether malignant-cell antigen state and myeloid/stromal contexture interact clinically, while leaving the direction and mechanism of that relationship unresolved.

## 5 Hypothesized links between myeloid signals and B-cell identity attenuation

No current clinical study has directly demonstrated that myeloid-derived signals induce coordinated loss of B-lineage antigens in DLBCL. Three non-mutually exclusive hypotheses can nevertheless be tested: selection, induction and spatial protection.

The selection hypothesis proposes that rare B-cell-identity-low or antigen-low malignant cells pre-exist before therapy. A suppressive or stromal-rich niche could increase their relative fitness or protect them during immune pressure, allowing expansion without requiring the niche to create the state.

The induction hypothesis is more speculative. Macrophage-, M-MDSC- and stromal-derived inflammatory or contact-dependent signals, including IL-6-associated pathways, provide biologically plausible inputs to malignant B cells (14, 32). Persistent IL-6/JAK/STAT3, IFN/NF-κB or stress signaling could, in principle, alter transcriptional or chromatin programs in malignant B cells. However, direct evidence connecting these signals to coordinated attenuation of CD19, CD20 and CD22 in CAR T-cell-resistant DLBCL is lacking. Candidate transcriptional and chromatin mechanisms should therefore be tested by perturbation and longitudinal profiling rather than positioned as established intermediates between a myeloid-rich niche and B-cell identity attenuation.

**Figure 1:**
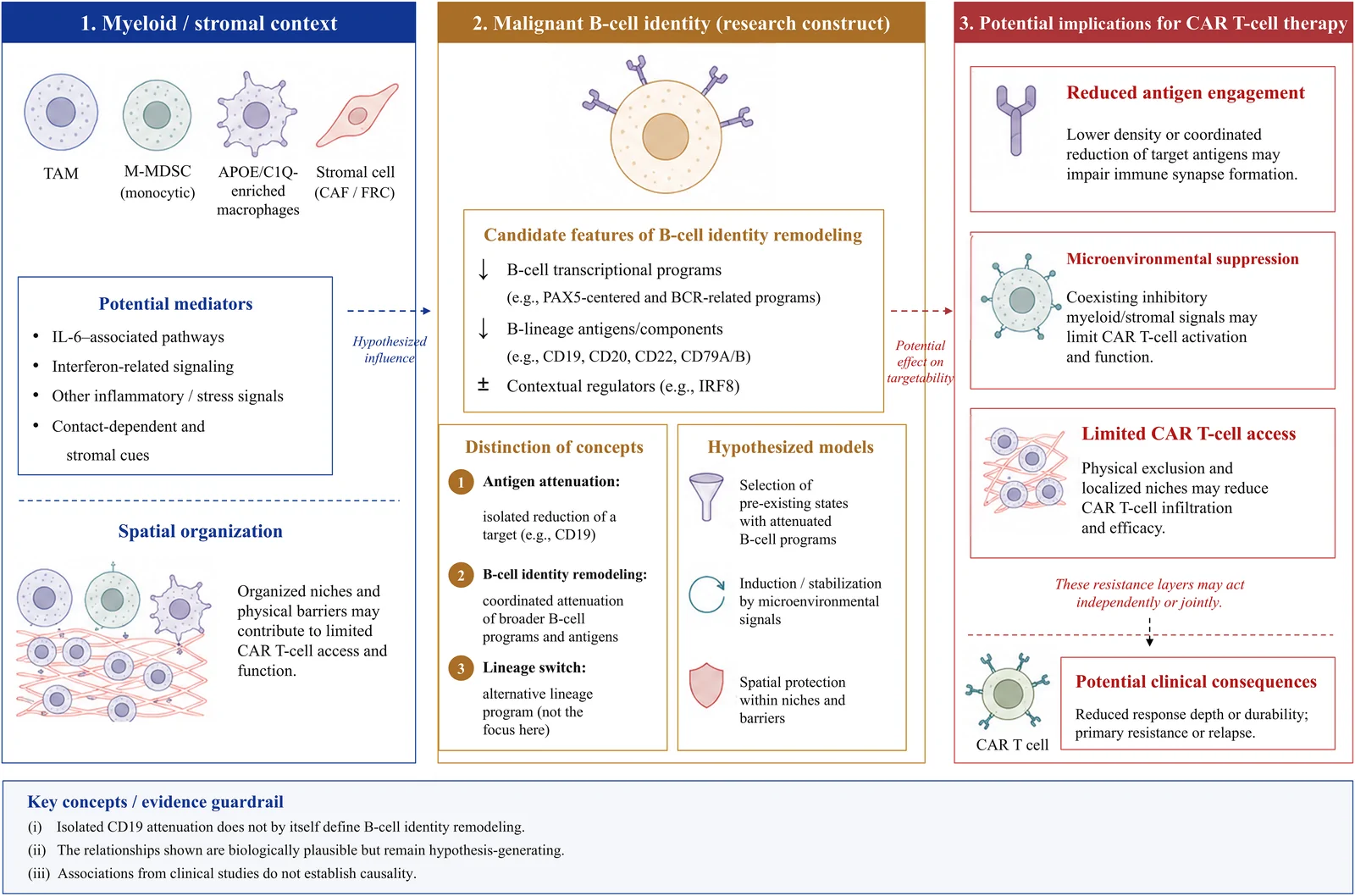
Hypothesis-generating framework linking myeloid/stromal context, B-cell identity remodeling, and potential CAR T-cell resistance. The schematic distinguishes antigen attenuation, broader identity remodeling, and lineage switch, and presents selection, induction/stabilization, and spatial protection as non-mutually exclusive hypotheses. Dashed relationships indicate biologically plausible but unproven links; clinical associations do not establish causality. TAM, tumor-associated macrophage; M-MDSC, monocytic myeloid-derived suppressor cell; CAF, cancer-associated fibroblast; FRC, fibroblastic reticular cell; BCR, B-cell receptor.

The spatial-protection hypothesis does not require alteration of tumor-cell identity. Myeloid- or stromal-rich regions may limit CAR T-cell access, increase inhibitory signaling or alter chemokine gradients, thereby protecting malignant cells with marginal antigen density during a critical cytotoxic window. Longitudinal spatial studies will be required to determine whether such niches merely coexist with antigen-low states, select for them, or actively stabilize them. These three non-mutually exclusive hypotheses and their proposed relationships are summarized in **Figure 1**.

## 6 Biomarker development: discovery, translational testing and future clinical implementation

Biomarker development should extend beyond prognostic prediction to support hypothesis-driven trial enrichment. Mechanistic discovery, prospective translational testing and routine clinical implementation require different levels of complexity. For mechanistic discovery, paired pretreatment, early on-treatment and relapse biopsies could integrate single-cell RNA sequencing, spatial transcriptomics or multiplex imaging with genomic, chromatin and methylation assays. This comprehensive workflow is intended to test directionality and compartment-specific biology rather than to serve as a routine clinical pathway.

A more feasible prospective translational panel could prioritize pretreatment formalin-fixed paraffin-embedded tissue, pathology-guided tumor-rich regions, CD19/CD20/CD22 and PAX5 protein assessment, and a limited myeloid or stromal readout. Targeted RNA profiling, fresh-tissue flow cytometry or multiplex imaging could be added when tissue and study resources permit. If a B-cell GES is used, its gene composition, platform, normalization and scoring algorithm should be prespecified rather than assumed to be transferable across assays. Bulk B-cell signatures require particular caution because reduced signal may reflect lower malignant-cell expression, variation in tumor purity or changes in nonmalignant B-cell abundance. At present, a routine clinical assay cannot yet be defined because no assay configuration or cutoff has been prospectively validated for treatment selection.

Accordingly, the four states defined by relative B-cell identity preservation/attenuation and myeloid/stromal enrichment in this framework should be regarded only as provisional trial-enrichment hypotheses.

## 7 Therapeutic hypotheses organized by antigen dependence and biological compartment

Therapeutic implications are clearer when strategies are organized by antigen dependence. Alternative-antigen approaches can bypass CD19 when another target remains expressed; CD20×CD3 bispecific antibodies such as glofitamab and epcoritamab are examples whose target engagement does not require tumor CD19 protein expression (33, 34). By contrast, CD19-directed agents such as loncastuximab tesirine and tafasitamab depend on tumor CD19 for target engagement and therefore do not bypass complete CD19 loss; no validated minimum tumor CD19 protein expression threshold has been established for treatment selection (35, 36). Multi-antigen CAR T-cell approaches, including CD19/CD20 and CD19/CD22 platforms, may reduce escape caused by isolated loss of one antigen (19, 20), but may not overcome coordinated attenuation of several B-lineage markers. The major therapeutic strategy classes, their antigen dependence, and key interpretive caveats are summarized in **Table 2**.

**Table 2:** Therapeutic strategies classified by antigen dependence and biological compartment.

Strategy class	Examples	Antigen dependence	Main interpretive caveat
Alternative-antigen bypass	CD20×CD3 bispecific antibodies; other non-CD19 targets	Does not require CD19 if the alternative target is retained	Alternative target expression remains necessary
Residual-CD19-dependent therapy	Loncastuximab tesirine; tafasitamab	Depends on tumor CD19 for target engagement	Does not bypass complete CD19 loss; no validated minimum tumor CD19 protein expression threshold for treatment selection
Multi-antigen targeting	CD19/CD20 or CD19/CD22 CAR T-cell platforms	Requires at least one targeted antigen to remain available	May not overcome coordinated attenuation of several B-lineage antigens
Tumor-cell antigen modulation	HDAC-inhibitor strategies in preclinical B-cell lymphoma models	Aims to increase target-antigen density	Evidence is preclinical and context-specific; not proven to restore full B-cell identity
Microenvironmental remodeling	Candidate macrophage/myeloid or IL-6/JAK/STAT3-directed interventions	Not primarily an antigen-replacement strategy	Requires biomarker and pharmacodynamic evidence of the targeted TME state
CAR T-cell-intrinsic fitness enhancement	Ex vivo decitabine priming of CAR T-cell products; other T-cell epigenetic strategies	Acts on the CAR T-cell product rather than tumor antigen expression	Do not interpret improved CAR T-cell fitness as tumor-cell antigen rescue

HDAC, histone deacetylase; TME, tumor microenvironment. Categories are research hypotheses and are not validated treatment-selection rules.

A distinct strategy is tumor-cell antigen modulation. Preclinical studies suggest that HDAC inhibition can sensitize selected B-cell lymphoma models to CAR T-cell-mediated killing (37, 38). More directly, chidamide has been reported to increase surface CD19 or CD22 expression in selected B-cell tumor models and enhance the activity of the corresponding CAR T cells (39, 40). These findings support pharmacologic modulation of individual target antigens, but do not establish restoration of a complete B-cell identity program or demonstrate clinical benefit from tumor-directed epigenetic priming.

Microenvironmental remodeling is a separate hypothesis. Potential myeloid- or inflammatory-axis interventions, including approaches directed at macrophage biology or IL-6/JAK/STAT3 signaling, would aim to alter the TME rather than replace a missing antigen (13, 14, 31, 32). Such combinations require biomarker, pharmacodynamic and safety endpoints because myeloid cells also contribute to antigen presentation, tissue homeostasis and inflammatory toxicity. They should not be added empirically without evidence that the relevant myeloid or inflammatory state is present.

**Figure 2:**
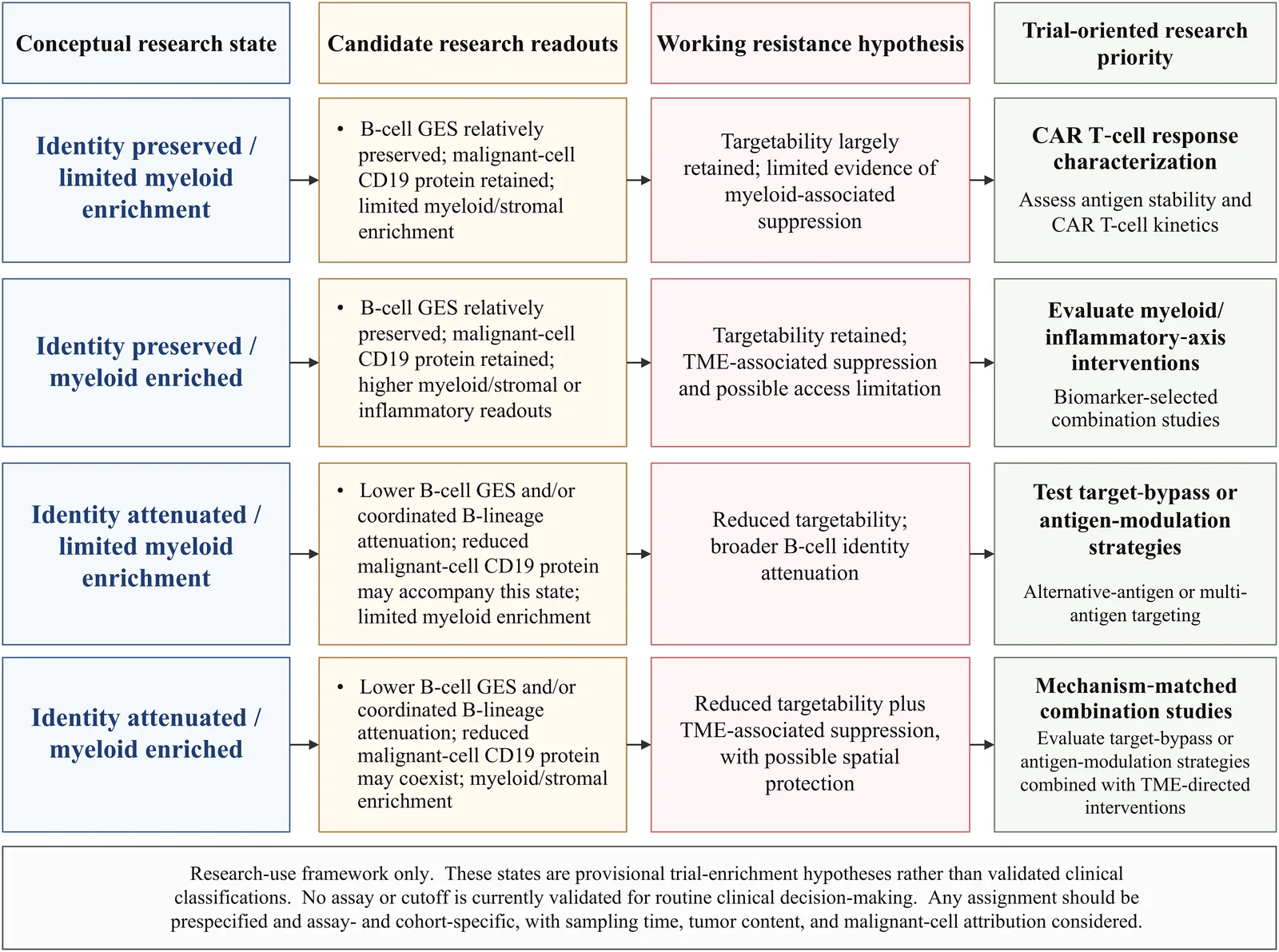
Translational research framework for provisional B-cell identity and myeloid-context states in CAR T-cell resistance. Four conceptual research states are organized by relative B-cell identity preservation/attenuation and myeloid/stromal enrichment, with candidate research readouts, working resistance hypotheses, and trial-oriented research priorities. These states are intended for hypothesis testing and prospective trial enrichment rather than validated clinical classification or routine treatment selection. No assay or cutoff is currently validated for assigning these states. GES, gene expression signature; TME, tumor microenvironment.

CAR T-cell-intrinsic fitness enhancement should also be kept distinct from tumor-cell antigen rescue. In the 2026 phase I/II study (NCT04697940), decitabine was used ex vivo during manufacturing of tandem CD19/CD20 CAR T-cell products; the reported biological effects included enrichment of memory-like progenitors, enhanced expansion and sustained persistence rather than restoration of lymphoma-cell surface CD19, CD20 or CD22 expression (19, 41). This provides clinical-translational support for CAR T-cell-intrinsic epigenetic modulation, consistent with broader epigenetic strategies to improve CAR T-cell function (42), but it should not be cited as evidence for reversal of B-cell identity remodeling. A trial-oriented framework integrating these conceptual states, candidate research readouts, working resistance hypotheses, and research priorities is summarized in **Figure 2**.

## 8 Discussion and future directions: testing association, induction and spatial protection

The central limitation of the proposed framework is that most supporting evidence is associative. A decisive study would require paired pretreatment, early on-treatment and relapse specimens from CAR T-cell-treated patients, with histology reported explicitly so that DLBCL-specific effects can be separated from broader LBCL findings. Single-cell profiling should define malignant and myeloid states, while spatial assays should test whether antigen-low malignant cells are physically enriched near specific myeloid or stromal populations.

Functional validation should then test directionality. DLBCL-macrophage or DLBCL-MDSC co-culture models could evaluate whether defined soluble or contact-dependent signals alter CD19, CD20, CD22 or PAX5 protein expression. Perturbation of IL-6/JAK/STAT3, IFN/NF-κB and selected chromatin regulators should be linked to antigen density, transcriptional state, chromatin accessibility and CAR T-cell killing. Organoid, tissue-slice or patient-derived models could add spatial structure and help distinguish induction from ecological protection.

Prospective trials should incorporate mechanism-matched translational endpoints. A trial evaluating tumor-directed antigen modulation before CAR T-cell infusion should measure target density before and after intervention. A trial combining CAR T-cell therapy with a myeloid-directed intervention should measure myeloid-state changes, T-cell infiltration, cytokine patterns and, where feasible, spatial redistribution. A trial designed to improve CAR T-cell-intrinsic fitness should instead track product phenotype, expansion, persistence and exhaustion-related states. Without compartment-matched endpoints, clinical activity alone would not establish the proposed mechanism.

## 9 Conclusion

CAR T-cell resistance in DLBCL is unlikely to be explained by a single compartment. LBCL clinical cohorts show associations among tumor-cell targetability, B-cell GES, immune contexture and myeloid/inflammatory features, while DLBCL single-cell and spatial studies demonstrate biologically organized tumor-immune ecosystems. These evidence streams are complementary but should not be conflated.

B-cell identity remodeling is therefore best viewed as a hypothesis-generating, multivariable research construct rather than a synonym for isolated CD19 loss. Its value is to connect broader malignant-cell lineage readouts with microenvironmental context while preserving the distinction between association, selection, induction and spatial protection. The immediate priority is to determine whether myeloid-rich niches merely coexist with antigen-low states, select for them or actively stabilize them. If prospective and functional studies support these relationships, the framework could inform biomarker-enriched trials of alternative-antigen targeting, tumor-cell antigen modulation, multi-antigen approaches, microenvironmental remodeling or CAR T-cell-intrinsic fitness enhancement.
